# Effect of COVID-19 on quality of life of persons aged >70 years with adult spinal deformity: A cross-sectional case-control study

**DOI:** 10.1097/MD.0000000000029954

**Published:** 2022-08-19

**Authors:** María Luz Suárez-Huerta, Alejandro Gomez-Rice, Miguel Carvajal Alvarez, Iria Carla Vazquez Vecilla, Enrique Izquierdo-Nuñez, Manuel Fernandez-Gonzalez, Lorenzo Zuñiga-Gómez, Jesus Betegon-Nicolas, Sonia Sanchez-Campos

**Affiliations:** a Institute of Biomedicine (IBIOMED), University of León, León, Spain; b Getafe University Hospital; c Ramon y Cajal University Hospital; d Cabueñes University Hospital; e León University Hospital; f Centro de Investigación Biomédica en Red de Enfermedades Hepáticas y Digestivas (CIBERehd), Instituto de Salud Carlos III, Madrid, Spain.

**Keywords:** adult deformity, COVID, lockdown, quality of life, spine surgery

## Abstract

This observational, cross-sectional case-control study evaluates the impact of coronavirus disease 2019 (COVID-19) on health-related quality of life (HRQoL) in elderly persons who have undergone surgery for adult spinal deformity (ASD). On December 31, 2019, the Chinese authorities first reported severe acute respiratory syndrome coronavirus 2, and on March 11, 2020, it was declared a pandemic. The pandemic seems to have had a negative effect on elderly patients who underwent ASD, in terms of functional and psychological quality of life. We selected patients with ASD aged > 70 years who had undergone surgery between 2010 and 2015 and compared them with age- and sex-matched patients who did not have ASD. We recorded sociodemographic variables, type of surgery, levels of spinal fusion, HRQoL (Scoliosis Research Society-22, Short Form 12 Health Survey, EuroQol-5D [EQ-5], Geriatric Depression Scale [Yesavage] [GDS], Modified Frailty Index-11, and Barthel index), fear of visiting a health center, fear of leaving one’s house, and adherence to preventive measures. The study population comprised 174 patients (mean [standard deviation] age, 77.3 [5.9] years; 86% women), of whom 87 had undergone surgery for ASD. The incidence of COVID-19 was higher in patients aged > 85 years (*P* = .041), urban areas (*P* = .047), and in patients in long-term care (*P* = .03). Similarly, no differences were observed for the ability to cope with the pandemic (*P* > .05). Patients who underwent surgery also had a higher risk of depression (GDS, 6.7 [*P* = .02]), a lower EQ-5 score (*P* = .001), a higher body mass index (*P* = .004), greater consumption of drugs (*P* < .001), especially opiates (*P* < .001). Patients who underwent surgery constitute a vulnerable population during the COVID-19 pandemic, with poorer quality of life and had a much higher risk of depression. They are also polymedicated and prefrail, adhere well to COVID-19 preventive measures, and do not seem to fear visiting health centers.

## 1. Introduction

The prevalence of adult spinal deformity (ASD) can reach 68% in elderly persons, with a negative impact on health-related quality of life (HRQoL) owing to associated pain and disability. The incidence of ASD is expected to rise gradually as the population ages.^[1]^

On December 31, 2019, the Chinese authorities first reported on a group of patients with respiratory infection of unknown cause that came to be known as severe acute respiratory syndrome coronavirus 2. The World Health Organization (WHO) subsequently termed it coronavirus 2019 disease (COVID-19), and on March 11, 2020, it was declared to be pandemic.^[2]^ When the present article is submitted, >323 million patients will have been infected and >5.5 million will have died, according to the WHO (January 18, 2022). These figures are unprecedented.^[3]^

Although COVID-19 affects persons of all ages, elderly people who have chronic diseases are especially vulnerable, with more severe clinical pictures and a higher mortality rate.^[4]^ Those living in long-term care facilities are particularly affected since they have been subject to considerable nosocomial transmission and frequent outbreaks.^[5]^

The quarantine periods generated a series of risks for mental health, leading to cases of depression, irritability, anxiety, fear, anger, and insomnia, together with the stress associated with loneliness and the fear of contracting the disease and not being able to say goodbye to loved ones. Moreover, many elderly persons do not have the necessary resources to cope with the stressful situation resulting from the COVID-19 pandemic. They lack material resources (e.g., no access to technology or basic necessities), social resources (e.g., few family members, or friends), and cognitive/biological resources (e.g., inability to use new technology or carry out daily activities).^[6,7]^

However, to date, the negative effects of the pandemic have not been reported in elderly patients who had undergone ASD surgery, in terms of functional and psychological quality of life.

The objective of this study was to determine how the pandemic influenced the functional and psychological status and HRQoL in patients who had undergone ASD surgery. Our hypothesis is that these patients, especially the older ones, have suffered a worse adaptation to the pandemic and will have a worse quality of life.

## 2. Methods

### 2.1. Study design

We performed an observational, cross-sectional case-control study of patients who underwent surgery for ASD in 2 tertiary hospitals (between January 2010 and December 2015) and compared our findings with those of patients without ASD from the same health care district.

The data were collected 2 times: before surgery and after surgery (between April and May 2020).

Of the 152 patients who underwent surgery for ASD, 134 fulfilled the inclusion criteria, 28 had died, 16 could not be contacted, and 3 refused to participate in the study. Finally, we included 87 patients who had undergone surgery. The characteristics of the patients and the type of surgery are summarized in Table 1.

**Table 1:** Characteristics of patients who underwent surgery.

Variable		(N = 87)	
Age, yr		77.3 (70–94)	
Female sex		75 (86.2%)	
Levels of spinal fusion		8.3 (5–17)	
Previous surgery		11 (36.8%)	
Subsequent surgical procedures		60 (69%)	
No. of procedures		2.3 (1–8)	
Infection		11 (12.6%)	
Site of fusion		Pelvis 39 (44.8%)	
		Lumbar-sacral 30 (34.5%)	
		Thorax 68 (78%)	
Type of osteotomy		1.57 (65.5%)	
		2.21 (24.1%)	
		3.9 (10.3%)	
ASA score		1.0 (0%)	
		2.53 (60.1%)	
		3.33 (37.1%)	
		4.1 (1%)	
Follow-up, yr		8.3 (5.4–10.2)	
MFI-11	0.16 (0–0.45)	Nonfrail	12 (13.8%)
		Prefrail	52 (59.8%)
		Frail	23 (26.4%)

ASA = American Society of Anesthesiologists; MFI-11 = Modified Frailty Index-11.

Our series comprised a total of 174 patients: 87 who underwent surgery for ASD and 87 who did not have ASD.

### 2.2. Ethical considerations

This study was approved by the institutional research ethics committee.

All patients gave their written informed consent to be included in the study.

### 2.3. Eligibility criteria

#### 2.3.1. Inclusion criteria.

##### 2.3.1.1. Case.

Includes patients who underwent surgery for ASD aged > 65 years, with spinal curvature in the coronal plane > 20°, a sagittal vertical axis > 5 cm, pelvic tilt > 25°, or thoracic kyphosis > 60°. The operated patients presented imbalance, with lumbar and radicular pain. All of them had received conservative treatment (pain killers, infiltrations, and rehabilitation).

##### 2.3.1.2. Control.

Includes patients without ASD aged > 65 years who visited a reference health center with other conditions between October and December 2019. These patients came to the health center for presenting stable chronic diseases that required follow-up, such as diabetes mellitus, dyslipidemia, or hypertension. These were matched by age and sex with those who underwent surgery.

#### 2.3.2. Exclusion criteria.

We excluded in both groups patients with tumors, infections or deformity (neuromuscular, congenital, or traumatic), and those who died.

##### 2.3.2.1. Case.

Patients who underwent surgery for ASD aged < 65 years, with spinal curvature in the coronal plane < 20°, a sagittal vertical axis < 5 cm, pelvic tilt < 25°, or thoracic kyphosis < 60°. Patients who still had conservative treatment options (pain killers, infiltrations, and rehabilitation) or did not want to undergo surgery.

##### 2.3.2.2. Control.

Patients with ASD aged < 65 years who visited a reference health center with acute, decompensated, or severe systemic diseases. Patients who were not matched for age and sex with those who underwent surgery.

### 2.4. Data collection

All information was collected by a researcher who did not participate in treatment.

We collected sociodemographic and clinical data, as follows: age, sex, body mass index (BMI), smoking history, and levels of spinal fusion (patients who underwent surgery).

All participants completed the EuroQol-5D (EQ-5), Geriatric Depression Scale (Yesavage) (GDS), Modified Frailty Index-11 (MFI-11), and Barthel Index Questionnaire (BI). The patients who underwent surgery also completed the following questionnaires: Short Form 12 Health Survey (SF-12), Scoliosis Research Society-22 (SRS-22), and Oswestry Disability Index (ODI).

Patients’ fear of visiting a health center, fear of leaving their house, and adherence to protective measures were assessed using a single-element scale ranging from 0 (not at all) to 10 (very much). These scales are inexpensive, valid, and reliable measuring instruments that can be used when assessment via other, more extensive scales is not possible.^[8,9]^

The EQ-5D is used to evaluate HRQoL. It consists of a descriptive system and a visual analog scale (EQ VAS). The descriptive system has 5 dimensions: mobility, self-care, usual activities, pain/discomfort, and anxiety/depression. Each dimension has 3 levels of severity (no problems, some problems, and severe problems). The EQ VAS is used to record the self-reported health status on a vertical VAS where the endpoints are labeled “The best health you can imagine” and “The worst health you can imagine.”^[10,11]^

The GDS is one of the most widely used instruments for identifying depression in elderly persons. It comprises 15 dichotomous questions (yes/no), of which 10 are positive and 5 are negative. A score ≥ 5 points indicates that the patient is at risk of depression, and a score > 10 points is considered an indicator of depression.^[12–14]^

The MFI-11 is a simplified form of the Canadian Study of Health and Aging Clinical Frailty Scale. It covers 11 variables or deficits: functional status, dependency, diabetes mellitus, lung problems, congestive heart failure, myocardial infarction, heart surgery, hypertension, previous transient ischemic attack, history of cerebrovascular accident, and peripheral vascular disease. The instrument yields an index ranging from 0 to 1 and classifies a patient as nonfrail (MFI = 0), prefrail (MFI > 0 and < 0.21), and frail (≥0.21).^[15,16]^

The BI is a generic measure of a patient’s level of independence with respect to basic activities of daily living (ADL). It assigns various scores and weights according to the patient’s ability to perform the activities. The 10 ADLs evaluated are feeding, moving from wheelchair to bed, personal toileting, getting on and off a toilet, bathing/showering, walking on a level surface, ascending and descending stairs, controlling bladder, and controlling bowel. The activities are scored as 0, 5, 10, or 15 points, with an overall range of 0 (completely dependent) to 100 (completely independent).^[17]^

### 2.5. Statistical analysis

The statistical analysis was performed using SPSS version 22.0. Statistical significance was set at *P* < .05. Descriptive statistics are expressed as mean and standard deviation. The inferential analysis was performed using the *t* test and Mann-Whitney test for continuous variables, the χ^2^ test for categorical variables, and the odds ratio (OR) with 95% confidence interval (CI).

## 3. Results

### 3.1. Epidemiological results

The study population comprised 174 patients (mean age, 77.3 [5.9] years; 86% women), of whom 87 had undergone surgery for ASD with a mean of 8.3 (3.5) levels of spinal fusion and 8.5 (2.2) years of follow-up.

Approximately 90% of patients had undergone osteotomy for correction of their deformity. According to the classification of Schwab et al,^[18]^ 57 patients were grade 1, 21 (23.3%) were grade 2, and 9 (10.3%) were grade 3 (Table 1).

### 3.2. Comparison between patients who underwent surgery and control group

**Figure 1.**
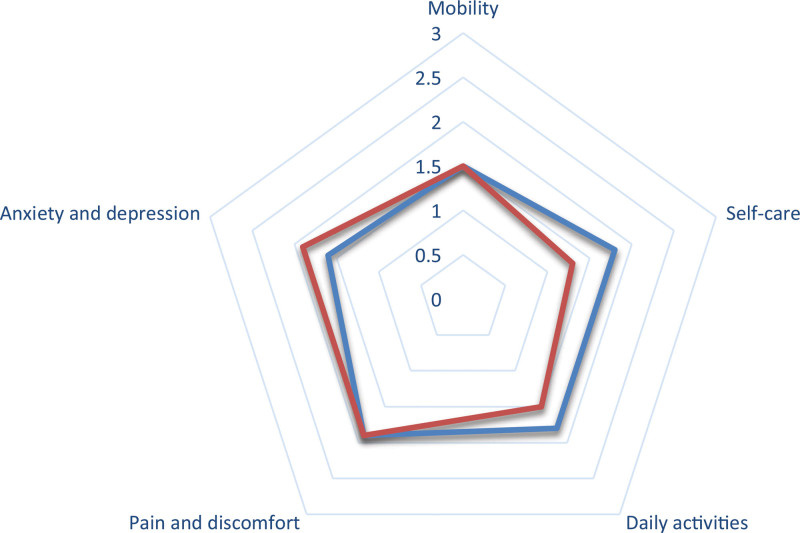
EuroQol-5D (EQ-5) in the study group (patients who underwent surgery) and control group (patients who did not undergo surgery).

No statistically significant differences in the incidence of COVID-19 were observed in patients who underwent surgery (*P* = .467); similarly, no differences were recorded in coping with the pandemic (*P* > .05), fear of visiting a health center (*P* = .693), fear of leaving one’s house (*P* = .325), or adherence to protective measures (*P* = .343). In contrast, we did observe a higher risk of depression, with a GDS of 6.7 (*P* = .02, OR = 2, 95% CI = 1.1–3.6) and a poorer score in the EQ-5 (*P* = .001, OR = 1.7, 95% CI = 1.2–3.2) (Fig. 1).

Patients who underwent surgery had a higher BMI (*P* = .004, OR = 1.9, 95% CI = 1.1–3.6), took more drugs (*P* < .001, OR = 2.9, 95% CI = 1.5–5.5), especially opiates (*P* < .001, OR = 3.3, 95% CI = 1.7–6.3), had a lower educational level (*P* = .004), and had significant differences in their place of residence (*P* < .001) (Table 2).

**Table 2:** Characteristics of the study group vs control group.

Variable	Study group	Control group	*P*	Odds ratio, N (CI 95%)
	Patients who underwent surgery for ASD (N = 87)	Patients who did not undergo surgery for ASD (N = 87)		
Age, yr	77.3 (70–94)	77.3 (70–94)	1	1
Sex				
Female	75 (86.2%)	75 (86.2%)	1	1
Male	12 (13.8%)	12 (13.8%)		
Residence				
Rural	21 (24.1%)	4 (4.6%)	<0.001	6.6 (2.1–20.1)
Urban	66 (75.8%)	83 (95.4%)		
COVID-19, N (%)	9 (10.3%)	12 (13.7%)	0.467	0.7 (0.2–1.7)
BMI	29.6 (15.62–45.9)	27 (16.65–42.81)	0.004	1.9 (1.1–3.6)
Educational level, N (%)				
None	14 (16.1%)	4 (4.6%)	0.004	–
Primary	50 (57.5%)	64 (73.5%)		
Secondary	16 (18.4%)	11 (12.6%)		
Higher	7 (8%)	8 (9.1%)		
Cohabitants, N (%)				
None	17 (19.5%)	21 (24.1%)	0.427	–
One	47 (54%)	52 (59.7%)		
≥2	16 (18.3%)	10 (11.6%)		
Institutionalized	7 (8%)	4 (4.6%)		
Clinical history/comorbid conditions				
No. of drugs	8.9 (2–22)	6.4 (0–26)	<0.001	2.95 (1.5–5.5)
Opiates, N (%)	45 (51.7%)	21 (24.1%)	<0.001	3.3 (1.7–6.3)
Psychiatric drugs, N (%)	28 (32.1%)	40 (45.9%)	0.071	0.6 (0.3–1.1)
Smoking, N (%)	6 (6.9%)	3 (3.4%)	0.2	1.8 (0.4–7.2)
Hypertension, N (%)	61 (70.1%)	62 (71.2%)	0.868	1.1 (0.6–2.2)
Heart disease, N (%)	25 (28.7%)	15 (17.2%)	0.071	2.4 (1.2–4.8)
Kidney disease, N (%)	6 (6.8%)	11 (12.6%)	0.237	0.5 (0.2–1.6)
Diabetes mellitus, N (%)	13 (14.9%)	13 (14.9%)	1	1.3 (0.6–2.8)
Obesity, N (%)	40 (45.9%)	22 (25.3%)	0.005	2.5 (2–4.7)
Lung disease, N (%)	6 (6.8 %)	12 (13.7%)	0.135	0.6 (0.2–1.5)
Mental disorder, N (%)	33 (37.9%)	39 (44.8%)	0.356	0.9 (0.5–1.5)
Cancer, N (%)	23 (26.7%)	22 (26.6%)	0.98	1.2 (0.6–2.4)
Osteoporosis under treatment, N (%)	23 (26.4%)	18 (20.6%)	0.009	2.4 (1.2–4.9)
Walking with help, N (%)	65 (74.7%)	20 (22.9%)	<0.001	10 (5.3–20.6)
Coping with the COVID-19 pandemic				
Fear of leaving one’s house (VAS)	4.28 (0–10)	3.79 (0–10)	0.325	1.4 (0.7–2.6)
Fear of visiting a health center (VAS)	4.6 (0–10)	4.88 (0–10)	0.693	0.9 (0.5–1.6)
Adherence to protective measures (VAS)	9.17 (4–10)	9.42 (6–10)	0.343	0.8 (0.4–1.5)
Quality of life				
GDS	6.67 (0–14)	5.23 (0–14)	0.022	2 (1.1–3.6)
Barthel index	90.81 (30–100)	96.25 (95–10)	0.871	0.6 (0.5–0.7)
EQ-5	0.45 (0.01–0.85)	0.58 (0.01–0.85)	0.001	1.7 (1.2–3.2)
Mobility	1.87 (1–3)	1.3 (1–3)	<0.001	13.8 (6.5–29.4)
Self-care	1.7 (1–3)	1.2 (1–3)	<0.001	5.2 (2.6–10.3)
Daily activities	1.9 (1–3)	1.46 (1–3)	<0.001	5.6 (2.9–10.9)
Pain and discomfort	2 (1–3)	1.8 (1–3)	0.048	1.6 (0.8–3.2)
Anxiety and depression	1.87 (1–3)	1.97 (1–3)	0.364	0.7 (0.4–1.4)
EQ-5 VAS	49.3 (0–100)	61.2 (0–100)	0.001	2.3 (1.2–4.2)
MFI-11 ≥ 0.21, N (%)	23 (26%)	17 (19.5%)	0.281	1.5 (1.2–3.1)

ASD = adult spinal deformity, BMI = body mass index, COVID-19 = coronavirus disease 2019, EQ-5 = EuroQol-5D, GDS = Geriatric Depression Scale, MFI-11 = Modified Frailty Index-11, VAS = Visual Analog Scale.

Slightly more than a quarter of the patients who underwent surgery (26%) were frail (*P* = .28), were afraid to visit a health center (*P* = .02), were older (*P* = .04), had fewer levels of spinal fusion (*P* = .017), had a higher BMI (*P* = .03), and usually lived with other persons (*P* = .013). An American Society of Anesthesiologists score of III was recorded in 56.5% of frail patients (*P* = .04).

Baseline SF-12 values were very much below the mean in patients who underwent surgery, with physical component summary (PCS) score of 23.2 ± 5.2 and mental component summary (MCS) score of 46.9 ± 14.5. Eight years after surgery, their situation continued to improve, with a PCS of 31 ± 10 and no variations in the MCS score (46.8 ± 13.6). The PCS score improved by 8 ± 10. Improvements were also recorded in the ODI, SF-physical component, and SRS-22 (pain, self-image, and satisfaction) (Table 3).

**Table 3:** SF-12, SRS-22, and ODI in patients who undergo surgery.

Variables	Presurgery	Postsurgery	Change	*P*
SF-12 physical	23.2 ± 5	31 ± 10	7.8	0.038
SF-12 mental	46.9 ± 14	46.8 ± 13	–0.1	0.46
SRS-total	2.3 ± 0.43	2.9 ± 0.7	0.3	<0.01
SRS-function	2.6 ± 0.5	2.9 ± 0.7	0.3	<0.01
SRS-pain	1.7 ± 0.59	2.6 ± .0.9	0.9	<0.01
SRS-self-image	2.37 ± 0.61	2.8 ± 0.8	0.43	<0.01
SRS-mental health	3 ± 0.9	3.2 ± 0.9	0.2	0.12
SRS-satisfaction	1.6 ± 1	2.9 ± 0.76	1.3	<0.01
ODI	51.4 ± 11	39.2 ± 15	–12.2	<0.01

ODI = Oswestry Disability Index, SF-12 = Short Form 12 Health Survey, SRS-22 = Scoliosis Research Society-22.

### 3.3. Characteristics of patients with COVID-19

Analysis of the COVID-19 group revealed incidence to be higher in patients aged > 85 years (*P* = .041), patients living in an urban area (*P* = .047), and patients living in long-term care facilities (*P* = .03) (Table 4).

**Table 4:** Characteristics of patients with and without COVID-19.

Variables	COVID+ (N = 21)	COVID− (N = 153)	*P*
Age, yr	78.23 (70–94)	77.04 (70–94)	0.474
Sex			
Female	16 (76.2%)	134 (87.6%)	0.16
Male	5 (23.8%)	19 (12.4%)	
Residence			
Rural	0 (0%)	25 (16.3%)	0.047
Urban	21 (100%)	127 (83%)	
Previous surgery	9 (42.9%)	78 (51%)	0.467
BMI	28.4 (19.8–40)	28.3 (15.6–45.9)	0.947
Educational level, N (%)			
None	0 (0%)	18 (11.8%)	0.059
Primary	19 (95%)	95 (61.8%)	
Secondary	2 (9.5%)	25 (16.4%)	
Higher	0 (0%)	15 (9.9%)	
Cohabitants, N (%)			
None	3 (14.3%)	35 (23%)	0.033
One	9 (42.9%)	90 (58.6%)	
≥2	5 (23.8%)	21 (13.8%)	
Institutionalized	4 (19%)	7 (4.6%)	
Clinical history/comorbid conditions			
No. of drugs	8.6 (0–22)	7.6 (0–26)	0.337
Opiates, N (%)	9 (45%)	57 (37.3%)	0.517
Psychiatric drugs, N (%)	11 (52.4%)	57 (37.5%)	0.199
Smoking, N (%)	2 (9.5%)	7 (4.6%)	0.451
Hypertension, N (%)	16 (76.2%)	107 (69.9%)	0.583
Heart disease, N (%)	6 (28.6%)	34 (22.5%)	0.549
Kidney disease, N (%)	3 (9.5%)	14 (9.2%)	0.865
Diabetes mellitus, N (%)	2 (9.5%)	24 (15.9%)	0.439
Obesity, N (%)	9 (42.9%)	53 (34.9%)	0.479
Lung disease, N (%)	3 (14.3%)	15 (9.9%)	0.549
Mental disorder, N (%)	11 (52.4%)	61 (39.9%)	0.286
Cancer, N (%)	5 (23.8%)	40 (26.1%)	0.638
Osteoporosis under treatment, N (%)	2 (18.2%)	39 (25.4%)	0.594
Walking with help, N (%)	10 (47.6%)	75 (49.3%)	0.882
Coping with the COVID-19 pandemic			
Fear of leaving one’s house (VAS)	4.42 (0–10)	3.9 (0–10)	0.588
Fear of visiting a health center (VAS)	5.57 (0–10)	4.63 (0–10)	0.274
Adherence to protective measures (VAS)	8.84 (6–10)	9.36 (4–10)	0.063
Quality of life			
GDS	6.4 (0–14)	5.89 (0–14)	0.598
Barthel index	91.87 (70–100)	91.11 (30–100)	0.893
EQ-5	0.52 (0.16–0.79)	0.52 (0.01–0.85)	0.938
Mobility	1.57 (1–2)	1.59 (1–3)	0.867
Self-care	1.57 (1–3)	1.45 (1–3)	0.433
Daily activities	1.76 (1–3)	1.69 (1–3)	0.666
Pain and discomfort	1.95 (1–3)	1.95 (1–3)	0.992
Anxiety and depression	1.76 (1–3)	1.94 (1–3)	0.288
EQ-5 VAS	57.38 (20–100)	55.57 (0–100)	0.728
MFI-11	0.16 (0–0.45)	0.15 (0–0.55)	0.668

BMI = body mass index; COVID-19 = coronavirus disease 2019, EQ-5 = EuroQol-5D; GDS = Geriatric Depression Scale; MFI-11 = Modified Frailty Index-11, VAS = Visual Analog Scale.

## 4. Discussion

Patients who are candidates for surgery to treat ASD are increasingly elderly owing to their longer life expectancy. They are, therefore, frailer and more frequently subject to complications.^[16,19–22]^

The unprecedented situation brought about by the COVID-19 pandemic has led us to face one of the strictest lockdowns in Europe and forced us to adapt quickly to a new lifestyle.^[23,24]^ This situation has had repercussions for all of us and for the patients we treat.

In the present study, we used generic HRQoL scales (EQ-5, SF-12, BI) and specific spinal scales (SRS-22 and ODI), a scale to assess depression in elderly persons (GDS), and a scale to assess frailty (MFI-11). Generic approaches were used in order to cover important common domains for the general population, since some patients did not have ASD, and specific approaches were used to evaluate the impact of ASD, depression, and frailty.^[25,26]^

Initial SF-12 values were very low in patients who underwent surgery for ASD. The mean SF-12 score in Spain for patients aged > 75 years is PCS 41.8 and MCS 49.8,^[27]^ whereas for patients in the present study, presurgery PCS was 23.2 ± 5.2 and presurgery MCS was 46.9 ± 14.5. Eight years after surgery, patients continue to improve, with a PCS score of 31 ± 10 and no variation in MCS (46.8 ± 13.6), very possibly because of the pandemic.

The results reported for the EQ-5 were similar to those of the Spanish National Health Survey (ENSE). Patients aged > 75 years have an EQ index of 0.7 and those who underwent surgery have an index of 0.4. They also have a poorer perception of their HRQoL (EQ-5 VAS) 62 vs 49. Therefore, the study population has very high levels of anxiety and depression, with an EQ-5 of 1.9, compared with an EQ-5 of 0.4 for the age-matched general population (ENSE data).^[28]^

In terms of functioning, our data are consistent with those of other studies, namely, lockdown did not negatively affect patients, as shown by the SRS-22 and SF-12.^[29]^ However, we cannot say the same for psychological status, since, although statistically significant differences were not recorded in the psychological dimension of SRS-22 or the SF-12, the GDS revealed that more than half of the study population is at risk of depression (GDS > 5), which was more frequent in women and patients who underwent surgery. This finding is most likely due to the strict lockdown measures.^[30,31]^ Although necessary, self-isolation generates a series of risks for mental health and increases the risk of depressive disorders, post-traumatic stress, and even cognitive impairment.^[32–36]^

The incidence of depression seems to be higher in patients who underwent surgery, although we are unable to state that spinal surgery is a risk factor. However, we do know that chronic pain increases the risk of depression up to 4-fold^[37,38]^ and that patients with depression may have a heightened perception of pain.^[39]^ They may also be more subject to surgical complications and at risk of further surgery.^[40,41]^

Mood disorders negatively affect functioning and quality of life. Some studies have shown an inverse correlation between depression and HRQoL and between depression and self-perception of health, with depression acting as a vulnerability factor. Consequently, we can state that depression and quality of life are closely associated. Similarly, we must not forget that ≈20% of patients with chronic diseases can eventually develop a major depressive disorder.^[42]^

Among patients who underwent surgery, we found no greater incidence of COVID-19 or differences in ability to cope with the pandemic in terms of fear of visiting a health center, fear of leaving one’s house, or adhering to protective measures. Similarly, we found no differences in levels of anxiety, which were high in both groups. Our results are comparable to those reported elsewhere.^[43]^ In addition, patients who underwent surgery tended to have greater BMI, greater risk of depression, more frequent consumption of drugs (including opiates), and a lower educational level, all of which probably contribute to their lower HRQoL.

We found consumption of drugs to be common, especially in patients undergoing surgery, who took a mean of 9 drugs per day. It is also noteworthy that 52% took opiates.

Polymedication is common in elderly patients and has become a public health problem in developed countries, not only because of its prevalence, which is around 70% in frail persons, or its consequences but also because it is increasingly frequent. Consequently, recent decades have seen an increase in the percentage of patients who are polymedicated, and this seems to be associated with aging of the population and increased morbidity in elderly persons. Polymedication in the elderly is independently associated with increased mortality, poorer HRQoL, and greater dependence. Therefore, it seems necessary to try to reduce consumption of drugs in persons for whom they may do more harm than good, particularly in frail elderly patients (both those who are institutionalized and those who live in the community), who could benefit from controlled deprescription.^[44]^ Polymedication affects HRQoL, especially in the case of opiates, which were consumed by more than half of the patients in this study. In addition, despite being very potent analgesics, opiates are associated with impaired functional capacity and an increased number of falls.^[45]^ Misuse of opiates is one of the main causes of accidental death. The United States Centers for Disease Control and Prevention reported that 25% of patients receiving long-term opiate therapy develop opiate use disorder, whose risk is associated with dose and duration of treatment.^[46]^

Although the incidence of COVID-19 was similar in both groups (10%–13%), we found no statistically significant differences between patients who underwent and did not undergo surgery. This may be because the type of patient was similar in both cases (prefrail), thus making them more susceptible to disease and leading to greater mortality.^[7,47]^ Elderly persons have been the most affected population to date, since the conditions they are affected by increase their frailty and leave them vulnerable to greater morbidity if they contract COVID-19.^[7,24]^

Biological age is not the same as chronological age. However, the prevalence of frailty increases with age, owing to factors such as the accumulation of health disorders. A study of almost 3000 elderly persons showed that frailty was more common with age and that at age 95 years, practically all patients in the sample were frail owing to an accumulation of health deficits.^[48]^

The residents of care homes have been considerably affected by COVID-19, since they are vulnerable patients with poorer HRQoL, poorer physical health (fecal incontinence, disability) and mental health (depression, dementia), and reduced opportunities for social relationships since many live alone or have few relatives.^[49–52]^ Very often, they live in social-health care centers, where staff change frequently, with many visitors, and resources are sometimes lacking, thus leaving them very vulnerable.^[2,5,24]^

We also recorded more cases in urban areas, as reported elsewhere, possibly owing to the high population density and the high level of local and international interconnectivity, which left patients particularly vulnerable to the spread of the virus. Approximately 90% of cases throughout the world have been in urban areas.^[53,54]^

We found that the frailest patients were the least frightened of visiting a health center, very probably because they see a health center as a safe, controlled place, where they feel protected, especially if they are aware of their frailty. Such patients seem to have a good family support structure since most live with other people and probably do not have to visit a health center alone, thus reducing their anxiety. In addition to these factors, it is worth noting that for many, telemedicine is not a readily available option owing to the scarce use they make of new technology and their low educational level.^[55,56]^

Our study findings are subject to various limitations. First, as the sample was small, our results should be confirmed in a larger series. Second, the study design was retrospective. Third, the sample was heterogeneous. These limitations affect the vast majority of studies because ASD has various causes.^[57,58]^ Fourth, as the study was limited to a single country, it is important to consider variations with other countries, where the pandemic has evolved differently.

Despite its limitations, ours is the first study to examine in depth the psychological and functional effects of the pandemic using specific scales with a control group comprising patients who did not undergo surgery. Therefore, we were able to evaluate in greater detail the impact of the pandemic on patients who underwent surgery for ASD.

## 5. Conclusions

Patients who underwent surgery constitute a vulnerable population during the COVID-19 pandemic, with poorer quality of life and had a much higher risk of depression.

They were also polymedicated and prefrail. They do not seem to be reticent about visiting a health center and adhere to protective measures.

It seems necessary to implement multidisciplinary units to attempt to improve the situation of elderly persons. These should diagnose and treat depression, reduce prescription of drugs, improve functioning, and provide support to frail elderly persons who live alone.

We should make every effort to maximize physiological and psychological status before and after surgery to improve the quality of life of elderly patients who undergo surgery for ASD.
